# Tso-Hsin Cheng: The founder of modern ornithology and zoogeography in China

**DOI:** 10.1007/s13238-020-00761-3

**Published:** 2020-07-13

**Authors:** Fumin Lei, Gang Song

**Affiliations:** Key Laboratory of Zoological Systematics and Evolution, Institute of Zoology, Chinese Academy of Sciences, Beijing, 100101 China

**Figure 1:**
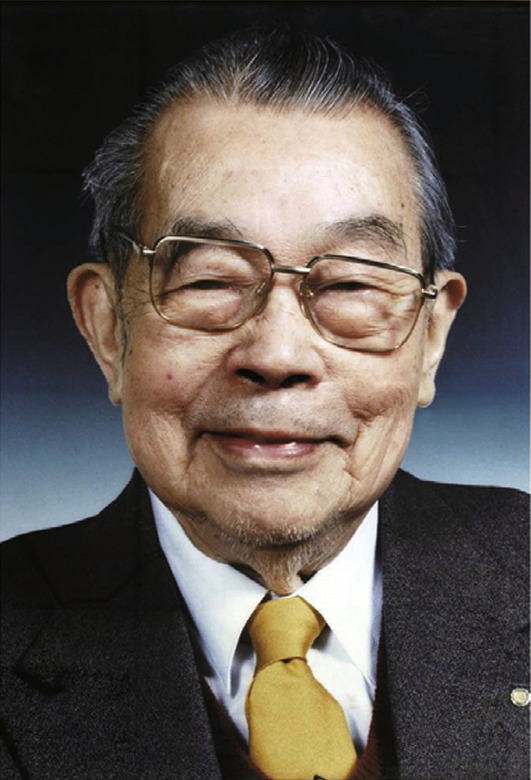
Cheng Tso-Hsin (1906–1998)

Tso-Hsin Cheng (Zuoxin Zheng, 郑作新) (Fig. 1) was born in Fuzhou, Fujian Province on November 18, 1906. He earned his Ph.D. from the University of Michigan in 1930, at the age of 23. After graduation, he returned to his homeland, where he devoted all his life to ornithology and zoogeography, and became a world-wide renowned scientist.

In the early time before the birth of the People’s Republic of China, ornithological studies in China were mainly carried out by western naturalists, focusing on scientific expeditions, species description and taxonomy. For example, the earliest bird checklist of China was published by Swinhoe, in the Proceedings of the Zoological Society of London in 1863, documenting 454 bird species in China. La Touche (1931–1934) published “A Handbook of the Birds of Eastern China”, in which a total of 750 species and subspecies of birds were descripted. However, they rarely focused on avian fauna and distributional concerns. Chinese scientists, such as Tsen-Hwang Shaw (Zhenhuang Shou, 寿振黄), Kwok-Yung Yen (Guorong Ren, 任国荣) and Tso-Hsin Cheng started ornithological research in the 1920’s. Prof. Cheng is the most accomplished scientist in Chinese ornithology. He started the first fieldwork on wild birds and their population characteristics in China, “A report for three years’ field survey (1938–1941) of birds in Shaowu”, covering multiple aspects of birds in that area, including their distribution, abundance and residential status. In 1947, he published the “Checklist of Chinese Birds” in the Transactions of the Chinese Association for the Advancement of Science, listing 1,087 species and 912 subspecies of birds in China (Cheng, 1947). This is the first checklist of birds in China with completed classification and distribution to be compiled by a Chinese ornithologist. Based on this checklist, Prof. Cheng published “On the geographical distribution of birds in China” in the same year in the journal China Science. This is the earliest comprehensive research on the species distribution and biogeography of birds in China, dividing the avian fauna into two zoogeographical realms (the Oriental realm and the Palearctic realm) and three zones (Mongolian Zone, North China Zone and South China Zone). This paper proposed a line beginning at the eastern edge of the Himalaya, along Qinling Mountains, Dabie Mountains, and across Yangtze River to the hilly areas in Fujian and Zhejiang as the boundary between the two realms. Prof. Cheng and Prof. Yung-tsu Chang (Rongzu Zhang, 张荣祖) then firstly proposed the concept of “Zoogeographical Regions of China” in 1956 (Cheng and Chang, 1956), which suggested two zoogeographical realms in China with Qinling Mountains as the boundary, and additionally divided the two realms into seven regions and sixteen sub-regions in more detail. This zoogeographical regionalization has been recognized worldwide as one of the basic frameworks of zoogeography of China. In 1987, Cheng published “A Synopsis of the Avifauna of China” (Cheng, 1987). This is an encyclopedic monograph with a substantial compiling of multiple aspects of birds in China. For each of the 1,186 species included in this monograph, nomenclature, breeding habitat, distribution and population status were also listed, which provided an inclusive ornithological reference for China, and is regarded as one of the classics of ornithological literature in the world.

Prof. Cheng is most famous for avian taxonomy and systematics. He and colleagues identified 16 new bird subspecies. They also studied the phylogeny and evolution of pheasants, laughing thrushes, and other bird families. After comparing the distribution and morphological variations of 14 subspecies of the silver pheasant (*Lophura nycthemera*), he found that more primitive subspecies were distributed at the periphery rather than the central area, and consequently proposed the “competitive exclusion” postulate to explain this pattern. This postulate is in accordance with the core idea of Darwin’s species competition concept, which is crucial for explaining the evolutionary history and distribution pattern of birds in China. Based on the well-archived taxonomy and distribution of birds, he led the publication of the Avian FAUNA SINICA series, and published the first volume (Volume 4, Galliformes) in 1978. He undertook great efforts in compiling the avian faunas, checklists, illustrations and a plethora of scientific reports about field expeditions across China. He made a tremendous contribution to ornithological and zoogeographical research with a number of fundamental reference works on species inventory and geographical distribution.

**Figure 2:**
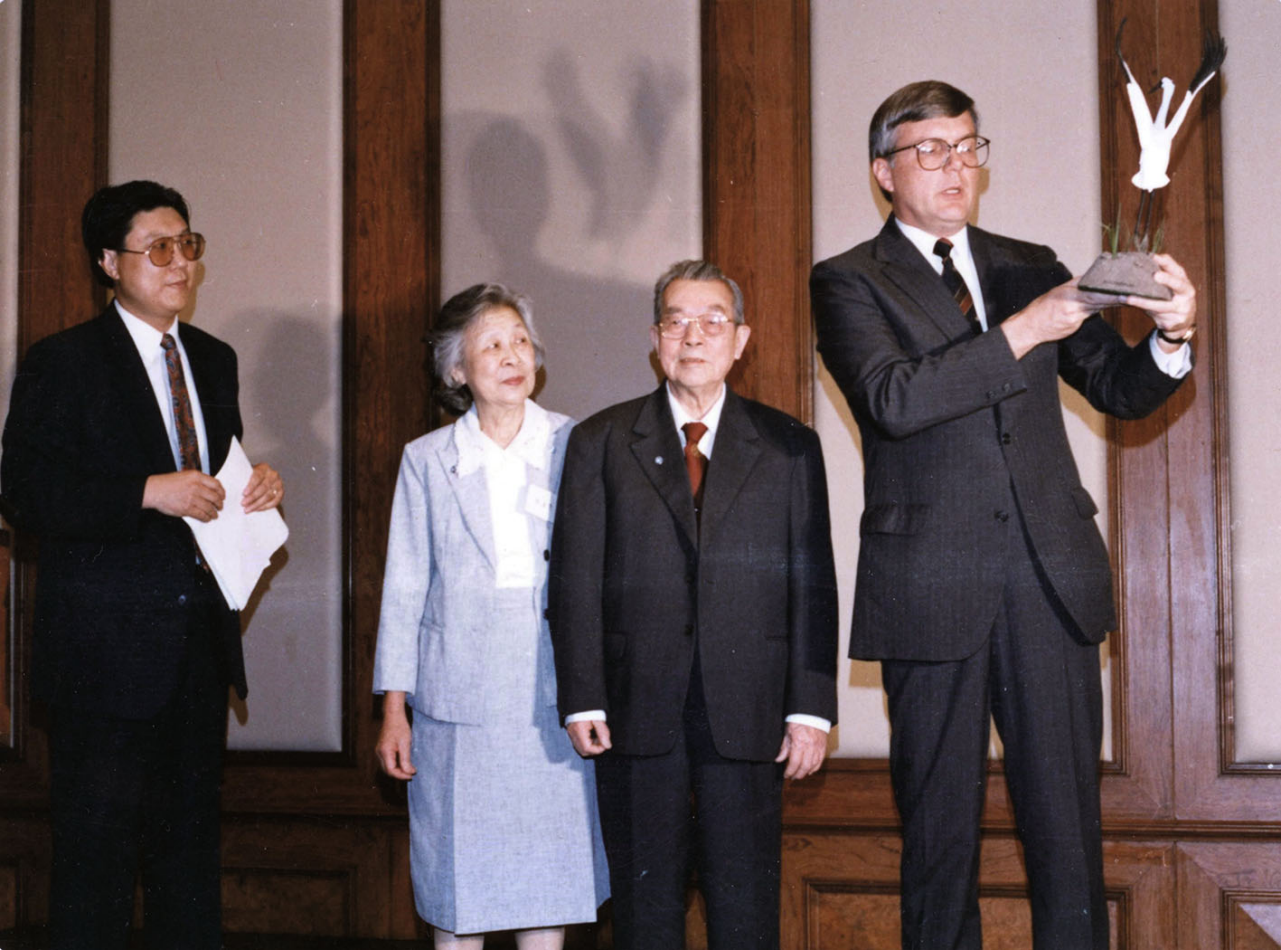
Cheng Tso-Hsin (second from right) was awarded the “Special Conservation Achievement Award”

Prof. Cheng was awarded many honors for his great scientific achievements in ornithology, such as the First Prize of Science and Technology Progress Award of the Chinese Academy of Sciences in 1989, the Second Prize of the National Natural Science Award in 1990, the Forestry Ministry Lifetime Honor Award for China Wildlife Protection in 1993, the Award of Choi Koon-Shum foundation for Chinese Academician of Sciences in 1995, and the Award of Qiu Shi Science & Technologies Foundation in 1996. In 1988, he was awarded the “Special Conservation Achievement Award” in international animal resources protection by the National Wildlife Federation (NWF) for his great contributions to the research and conservation of birds (Fig. 2). This is the first time that the NWF awarded a Chinese scientist outside of the United States of America.

**Figure 3:**
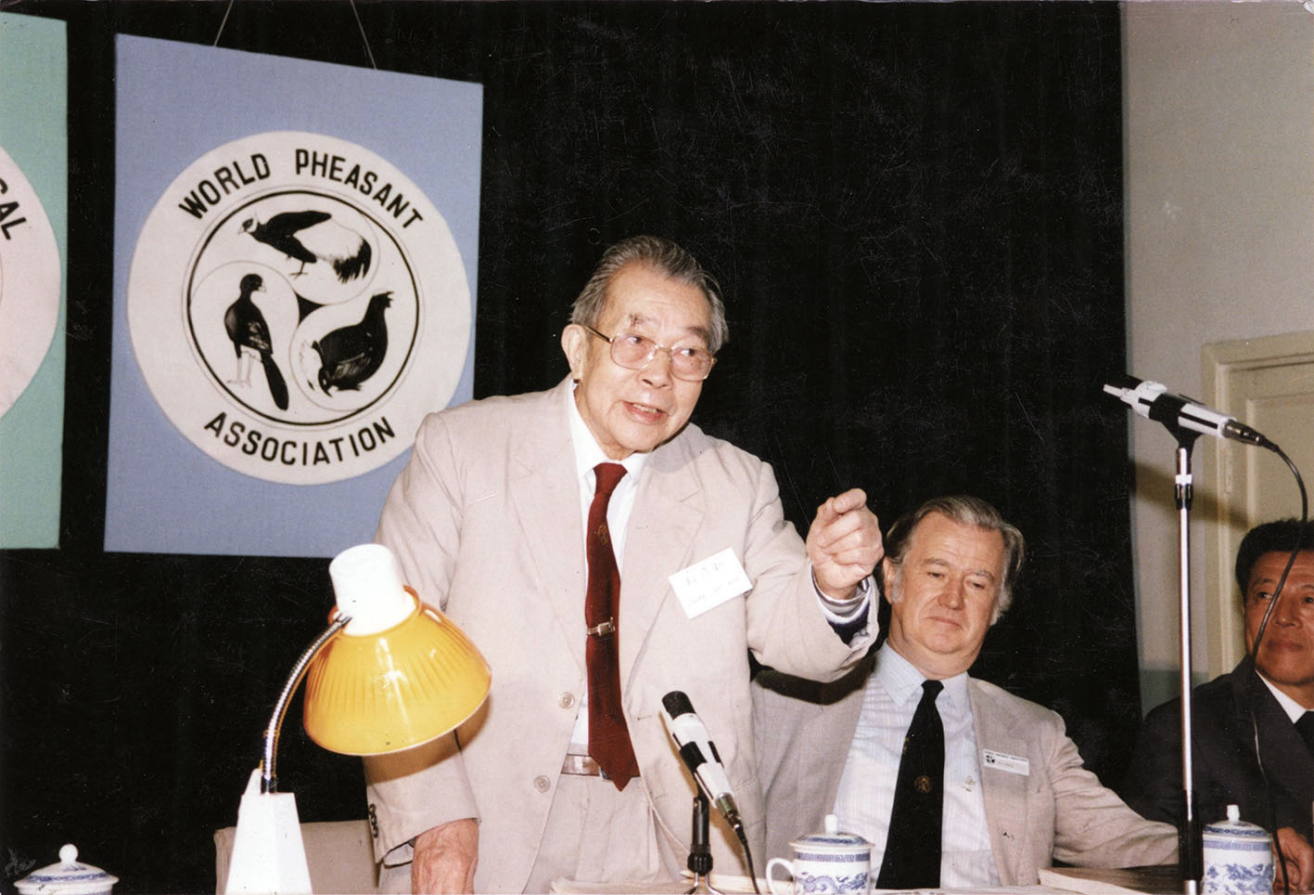
Cheng Tso-Hsin gave a welcome speech during the International Conference of World Pheasant Association in 1989

Prof. Cheng was elected as the vice-president, president, and the lifetime honorary president of the World Pheasant Association (Fig. 3). He was a consultant of the International Crane Foundation, and the honorary Chairman of the 22nd International Ornithological Conference. He is a cofounder of the China Zoological Society, in which he actively served as the general secretary, vice chairman, chairman, and honorary chairman. He is also a founder of the China Ornithological Society, in which he was elected as the first chairman, and later on the honorary chairman. In 1980, Prof. Cheng was elected a member of the Academia Sinica. In order to encourage more young students and scholars to pursue a career in ornithology, Prof. Cheng donated his prize money to set up the “Cheng Foundation for Ornithological Science” in 1994. To date, 28 awardees were honored, all of whom have become leading ornithologists in China, and some of them are leading international scientists.

Prof. Cheng passed away on June 27, 1998. To memorialize his great contributions to the development of ornithology in China and the world, Per Alström and Fumin Lei, collaborating with other colleagues, nominated a new bird species—*Locustella chengi*, in name of Prof. Cheng (Alström et al., 2015). His scientific merits and spirit in pursuit of science are being passed on between generations, illuminating the ornithological development in China.
